# Treatment before Diagnosis

**Published:** 1897-04-24

**Authors:** 


### TREATMENT BEFORE DIAGNOSIS.

So thoroughly is it inculcated into the student that the first and most important step in dealing with a case —that, in fact, on which all else hinges—is diagnosis, that it may seem rather like putting the cart before the horse to discuss the question what treatment should be adopted before the diagnosis is arrived at. Nevertheless, it is a matter of some importance to recognise that in practice treatment, and even perfectly correct treatment, in the early stage of a disease does not so entirely hinge on diagnosis as some theorists would have us believe, and that it often has to be undertaken before a diagnosis can be arrived at; while it unfortunately is a fact that treatment in that stage, if it be incorrect, may not only be useless for purposes of cure, and even destructive of all chance of cure, but, when it falls short of such efficacy for evil, may so mask the nature of the case as to render a usefully complete diagnosis quite impossible. It is for this reason that Mr. Greig Smith* has called attention to what he speaks of as the pre-diagnostic treatment of patients of whom the utmost that can be said at first is that they are suffering from some grave abdominal disease. There may be all the symptoms of shock together with abdominal pain, and it may be impossible to say whether the patient is suffering from colic, or acute obstruction, or perforation of some viscus, with intraperitoneal diffusion of the contents of a perforated stomach, of a peri-appendicular abscess, or of a tubal pregnancy with accompanying hæmorrhage. The question is what to do.

First of all, stuff must not be poured into the stomach till a diagnosis can at least assure us that the stomach is sound; and next, however tempting it may seem to produce narcosis by aid of the morphia syringe, it must be recognised that to do this is to render impossible all further steps in diagnosis short of abdominal section.

The first thing is to promote reaction. An ounce of brandy or whisky in about three ounces of milk should be given as an enema, the finger, at the moment of administration, making an examination of the rectum.

Then the patient is to be swathed in hot blankets and

* *Treatment*, March, 1897.

surrounded with hot-water bottles, and the medical man must then sit down and watch, making up his mind that he is not to leave his post until his diagnosis is complete—at least to this extent, that he shall have decided what is to be done.

He is not to be bullied by the anxiety of friends into declaring a premature opinion. He must watch the evolution of the symptoms, and, comparing them with his own store of knowledge as to the symptoms of the various conditions which might possibly give rise to the state of affairs before him, he is to make up his mind, bringing to his aid palpation, percussion, auscultation, and all those indications which the patient's own actions, or want of actions, give in the elucidation of the case. Then chloroform may be given, by which, in practice, colic may be eliminated. At any rate in the course of half an hour, by careful observation, and watching the natural evolution of the symptoms, a diagnosis should be arrived at so far, at least, as concerns the question whether to operate or not. The points on which Mr. Greig Smith insists are: To avoid morphia; to give nothing by the mouth; to give an enema containing a full dose of alcohol; to apply heat to the body; to sit down and watch, and not to leave the patient's side till reasons have been found for a definite line of treatment.

